# Effect modification by workplace social capital on the association between depression and workplace and family stress: the Japanese civil servant study

**DOI:** 10.1186/s12889-021-10767-z

**Published:** 2021-04-14

**Authors:** Nobue Nakahori, Michikazu Sekine, Takashi Tatsuse, Masaaki Yamada

**Affiliations:** 1 Faculty of Nursing Science, Tsuruga Nursing University, 78-2-1 Kizaki, Tsuruga, Fukui, 914-0814 Japan; 2 Department of Epidemiology and Health Policy, School of Medicine, University of Toyama, 2630 Sugitani, Toyama, Toyama 930-0194 Japan

**Keywords:** Depression, Gender, Japanese civil servants study, Workplace social capital

## Abstract

**Background:**

Mental health problems among workers have become an issue in Japan. The working environment for civil servants is becoming excessively stressful, and there is a need to prevent the onset of depression. In addition to stress at the workplace and at home, social capital has been reported as a factor associated with depression. This study examined whether workplace social capital reduces the association between depression and work-related stress or depression and home-related stress.

**Methods:**

A total of 3015 Japanese civil servants (1867 men and 1148 women) from Toyama Prefecture were included in this study. Data on depression and workplace social capital, work status, work stress, work–life balance, and physical health were collected.

**Results:**

The odds ratio for depression was higher for both men and women with low workplace social capital. For those with low workplace social capital, the adjusted odds ratio for depression was 2.93 (95% confidence interval [CI], 2.16–3.98) for men and 2.46 (95% CI, 1.74–3.49) for women. After adjusting for workplace social capital, the associations between depression and low job position, low job support, and moderate family–to–work conflict declined in men and were no longer significant. For women, the strength of the association between depression and unmarried status along with moderate control at work decreased and also lost significance. When the ORs for depression were stratified by high and low workplace social capital and compared with the ORs before stratification, the ORs for depression of long working hours and work–to–family conflict increased for both men and women in the low workplace social capital group.

**Conclusions:**

Workplace social capital mitigated the effect of workplace and family stress leading to depression in both men and women.

## Background

Suicide is the leading cause of death among workers in their twenties and thirties, second among those in their forties, and third or fourth among those in their fifties [1]. About 97% of suicides were reported to be due to a diagnosed mental disorder [2], indicating that mental health issues are a significant factor in suicide among workers. About 20% of workers annually take a leave of absence or resign due to mental health issues [3].

The work environment for civil servants is becoming increasingly difficult. Salaries are steadily declining compared to several years ago, the number of new hires is being scaled back, and positions vacated by retirees are being cut. At the same time, the workload is becoming more diverse and complex and the systems and public services that are in place are rarely reduced, meaning that the amount of work per person increases every year.

Under these circumstances, more and more civil servants suffer physical and mental health problems, such as depression, which can lead to suicide. Long-term sick leave among civil servants in 2019 was 2700 out of 100,000, of which 60% was due to “mental and behavioral disorders” [4]. As the size of the civil service workforce declines through attrition due to retirement, resignations, or suicides due to mental health issues, the increased stress on public services can affect the quality of services offered and the quality of life of the residents. It is a vicious cycle, increasing the workload on those workers who remain and negatively affecting the productivity of the entire workplace.

In order to avoid this cycle, it is important that mental health problems among workers are recognized as early as possible and efforts made to address the situation before it results in serious consequences. Previous studies have reported additional causes of depression among workers beyond overwork, including job stress, impaired work–life balance, and physical health [5]. Other reported causes of workplace depression include unpleasant bosses, unequal work environment [6–8], and dissatisfaction with work [9, 10]. Similarly, some people are able to work in busy departments and maintain their mental health and others are not, despite similar workloads. When we considered these differences, we wondered if social capital in the workplace, which can be described as good teamwork, had an effect in addition to personal qualities. There are several types of social capital, which reportedly to enhance physical and mental health as well as work engagement [11–14]. In order to maintain mental health, we thought that workplace social capital (defined in this study as understanding and acknowledging each other in the workplace) would be important.

Low workplace social capital has been reportedly associated with mental health issues around the world [15–19]. However, few have examined whether workplace social capital is independently associated with depression after adjusting for work–life condition, job stress, work–life balance, and physical health. There is a lack of research evaluating the effect modification of whether workplace social capital reduces the association between depression and these stressors. It is also important to understand how gender differences in physical and mental health [20–23] and disparities in sleep duration impact an individual’s response to these stressors and how workplace social capital can change the association between these stressors and depression [24, 25].

This study examined whether workplace social capital reduces the association between depression and work–life condition, job stress, work–life balance, and physical health, accounting for gender differences.

## Methods

This study has a cross-sectional study design.

### Study subjects

The Japanese Civil Service Study is an international collaboration with the British Civil Service Study (Whitehall Study) and has been conducted regularly since 1998 [24]. The subjects were all civil servants in 1 prefecture in central Japan. A total of 3997 persons responded to a questionnaire survey conducted in 2014. After excluding those who were not between the ages of 20 and 59 years old and those who had 1 or more missing responses to the survey items required for analysis, a total of 3015 respondents (1867 men and 1148 women) were included in the final analysis. The reason for excluding individuals in the age groups under 20 and over 60 is that they are small in number, and those over 60 may have different work experience, such as re-employment after retirement.

### Measures

#### Questionnaire

The items in the questionnaire were selected primarily from the Whitehall Study and reviewed by Whitehall researchers. The survey items included demographics (age and gender), depression (CES-D scores), workplace social capital, work–life condition (marital status, job position, work hours, shift work), job stress (control at work, demand at work, job support), work–life balance (family–to–work conflict, work–to–family conflict), and physical health (sleep duration and presence of longstanding illness). Variables other than workplace social capital were selected because a previous study on the same subjects [5] reported a significant association with depression.

Work–life condition included marital status, job position, work hours, and shift work. Marital status was categorized as “married” or “unmarried”; job position was categorized as “low grade,” “middle grade,” or “high grade”; work hours was categorized as “<9 hours ,” “9–11 h,” or “> 11 h”; and shift work was categorized as “yes” or “no.”

Karasek’s model [26] of job discretion, job demands, and job support was used as a rating scale for job stress. The model consisted of 25 items (15 items on control at work, 4 on job demands, and 6 on social support), and responses were selected from 4 options (common, sometimes, rarely, and never). Control at work was investigated with the following 15 items: (1) Do you have a choice in deciding how you do your job? (2) Do you have a choice in deciding what you do at work? (3) Others make decisions concerning my work. (4) I have a good deal of say in decisions about work. (5) I have a say in my own work speed. (6) my working time can be flexible. (7) I can decide when to take a break. (8) I have a say in choosing who I work with. (9) I have a great deal of say in planning my work environment. (10) Do you have to do the same thing over and over again? (11) Does your job provide you with a variety of interesting things? (12) Is your job boring? (13) Do you have the possibility of learning new things through your work? (14) Does your work demand a high level of skill or expertise? (15) Does your job require you to take the initiative? Job demands was investigated with the following 4 items: (1) Do you have to work very fast? (2) Do you have to work very intensively? (3) Do you have enough time to do everything? (4) Do different groups at work demand things from you that you think are hard to combine? Social support was investigated with the following 6 items: (1) How often do you get help and support from your colleagues? (2) How often are your colleagues willing to listen to your work-related problems? (3) How often do you get help and support from your immediate superior? (4) How often is your immediate superior willing to listen to your problems? (5) Do you get sufficient information from line management (your superiors)? (6) Do you get consistent information from line management (your superiors)? Responses are scored from 0 (often) to 3 (rarely), with higher total scores denoting more stressful situations. The total score was divided into 3 quartiles for each of the discretionary, demanding, and supportive aspects of the work, and then divided into 3 groups: “low,” “medium,” and “high.”

The Family-Work Conflict questions were used to assess work–life balance, consisting of 8 items, 4 for Family–to–work conflict and 4 for Work–to–family conflict [27]. Responses were selected from 3 options (not at all, some, and often). Family–to–Work conflict was investigated with the following 4 items: (1) Family matters reduce the time you can devote to your job. (2) Family worries or problems distract you from your work. (3) Family activities stop you from getting the amount of sleep you need to do your job well. (4) Family obligations reduce the time you need to relax or be yourself. Work–to–family Conflict was investigated with the following 4 items: (1) Your job reduces the amount of time you can spend with the family. (2) Problems at work make you irritable at home. (3) Your work involves a lot of travel away from home. (4) Your job takes so much energy you do not feel up to doing things that need attention at home. Responses were scored from 0 (never) to 2 (frequently), with higher total scores denoting situations with higher conflicts.

In terms of physical health, sleep duration was categorized as “< 6 h,” “6–8 h,” and “≥9 h,” and longstanding illness was measured by asking the respondents to answer the question, “Do you currently have a chronic disease or illness?” with two choices, “yes” or “no.”

#### Depression score

The Center for Epidemiologic Studies Depression Scale (CES-D) was used as a measure of depression and is a highly valid and reliable test [28]. The CES-D consists of 20 items, of which 16 are negative—denoting the presence of symptoms of depression, such as depressive mood, physical symptoms, and interpersonal relationships—and 4 are positive—denoting the absence of symptoms of depression, such as positive mood. Respondents were asked to rate, on a scale of 1 to 4, how frequently they experienced symptoms over the past week: none (1 point), 1–2 days (2 points), 3–4 days (3 points), or ≥ 5 days (4 points). On the CES-D, a total score of ≥16 suggested the presence of depression; however, our study used a score of ≥19 as suggestive of “depression” and ≤ 18 as “not depressed,” based on previous studies of Japanese civil servants [29].

#### Workplace social capital

Workplace social capital refers to 3 items in the standard version of the New Brief Job Stress Questionnaire [30]: “In our workplaces, we are willing to work together”; “In our workplaces, we understand and acknowledge each other”; and “In our workplaces, we are able to share work-related information.” In the short version, workplace social capital refers to the item “In our workplaces, we understand and acknowledge each other.” Responses are selected from 4 options: strongly agree (1 point), agree (2 points), disagree (3 points), or strongly disagree (4 points). In the current study, a shortened version was adopted, and respondents were classified as “high” if they answered “strongly agree” or “agree” to the question of whether they had social capital in the workplace, and “low” if they answered “disagree” or “strongly disagree.” The standard version of the New Brief Job Stress Questionnaire and its short version have been validated for reliability and validity [31, 32].

### Statistical analysis

The proportional differences between subjects’ characteristics were compared on the basis of gender using the Chi square (χ^2^) test. Age-adjusted Mantel–Haenszel tests were conducted to examine the relationship between workplace social capital and each variable, and the results were compared for each gender. In order to examine the association between workplace social capital and depression-related factors, the proportion of each depression-related factor in the high and low workplace social capital groups was compared on the basis of gender using the χ^2^ test. Logistic regression analyses were conducted for both genders, with the presence of depression as the dependent variable and each item as an independent variable. Several multivariate models [33] were constructed to examine how workplace social capital affects the association between depression and work–life condition, job stress, work–life balance, and physical health. The age-adjusted OR for depression was calculated first for each variable, after which the remaining variables (work–life condition, job stress, work–life balance, and physical health) were adjusted in order to calculate the OR and 95% CI, respectively, between depression and each variable. Effect modifications by workplace social capital on the association between depression and each variable was examined. The age-adjusted ORs for depression were calculated by logistic regression analysis, and the ORs of the subjects before stratification and the high and low groups after stratification by workplace social capital were compared. SPSS ver.23 was used for all analyses, with 5% being the significance level.

## Results

Table 1 shows the characteristics of the subjects according to gender. The mean age was 44.95 (SD 9.56) years for men and 38.30 (SD 10.70) years for women. The mean CES-D score was 13.50 (SD 8.39) for men and 15.39 (SD 8.75) for women. The mean workplace social capital was 2.98 (SD 0.68) for men and 2.91 (SD 0.68) for women. There was a significant difference between men and women in all variables. Working hours were longer for women and a higher percentage of women worked in shifts. Men were more likely to have more control at work, and women were more likely to have high job demands. Men were less likely to be supported at work. Family-to-work conflict and work–to–family conflict were higher for women. Women were more likely to sleep < 6 h and men were more likely to have longstanding illnesses. A higher percentage of women reported having little or no workplace social capital. Women were more likely to have higher levels of depression.

**Table 1:** Characteristics of subjects by gender

	Men(*n* = 1867)	Women(*n* = 1148)	χ^2^*p*-value
	%	%	
Age			< 0.001
20–29	9.2	26.8	
30–39	17.3	28.3	
40–49	36.8	25.0	
50–59	36.7	19.9	
Marital status			< 0.001
Married	80.2	55.7	
Unmarried	19.8	44.3	
Job position			< 0.001
Low grade	56.0	86.4	
Middle grade	26.4	10.7	
High grade	17.6	2.9	
Work hours			< 0.001
< 9 h	65.1	56.2	
9-11 h	26.4	34.6	
>11 h	8.5	9.2	
Shift work			< 0.001
Yes	8.1	43.9	
No	91.9	56.1	
Control at work			< 0.001
Low	26.6	33.0	
Intermediate	32.1	35.9	
High	41.3	31.1	
Demand at work			< 0.001
Low	34.3	28.1	
Intermediate	33.2	30.9	
High	32.5	40.9	
Support at work			< 0.001
Low	27.2	21.4	
Intermediate	37.9	35.1	
High	34.9	43.5	
Family–to–work conflict			< 0.001
Low	47.2	43.6	
Intermediate	15.5	12.2	
High	37.2	44.3	
Work–to–family conflict			< 0.001
Low	32.2	24.0	
Intermediate	36.6	34.5	
High	31.1	41.6	
Sleep hours			< 0.001
<6 h	15.2	20.6	
6-8 h	84.1	78.5	
≧9 h	0.7	0.9	
Longstanding illness			< 0.001
Yes	36.5	24.0	
No	63.5	76.0	
Workplace social capital			0.005
High	82.0	77.8	
Low	18.0	22.2	
Depression			< 0.001
Yes	23.0	30.5	
No	77.0	69.5	

Table 2 shows the association between depression and age-adjusted workplace social capital by gender. The variables that were significantly associated with lack of workplace social capital among men were low job position, low control at work, high job demands, low job support, high family–to–work conflict, high work–to–family conflict, and the presence of longstanding illness. The variables that were significantly associated with lack of workplace social capital among women were unmarried status, low control at work, high work demands, low job support, high work–to–family conflict, high family–to–work conflict, and less sleep (< 6 h).

**Table 2:** Age-adjusted workplace social capital differences in depression related factors by gender

		Workplace social capital		
	Depression-related factors	High(%)	Low(%)	χ^2^ *p*-value
Men (*n* = 1867)		(*n* = 1531)	(*n* = 336)	
Marital status	Unmarried	19.7	20.5	0.490
Job position	Low	54.2	64.3	< 0.001
Work hours	Long (>9 h)	34.9	34.8	0.894
Shift work	Yes	7.8	9.5	0.300
Control at work	Low	23.6	40.2	< 0.001
Demand at work	High	30.6	41.1	< 0.001
Support at work	Low	21.5	53.3	< 0.001
Family–to–work conflict	High	33.9	52.4	< 0.001
Work–to–family conflict	High	28.2	44.6	< 0.001
Sleep hours	Short (<6 h)	14.4	18.8	0.079
Longstanding illness	Yes	35.5	41.4	0.031
Women(n = 1148)		(*n* = 893)	(*n* = 255)	
Marital status	Unmarried	42.9	49.0	0.002
Job position	Low	86.0	87.8	0.273
Work hours	Long (>9 h)	43.0	46.7	0.311
Shift work	Yes	43.0	47.1	0.148
Control at work	Low	28.0	50.6	< 0.001
Demand at work	High	38.9	48.2	0.010
Support at work	Low	14.3	46.3	< 0.001
Family-to-work conflict	High	42.8	49.4	0.167
Work–to–family conflict	High	38.2	53.3	< 0.001
Sleep hours	Short (<6 h)	18.4	28.6	0.001
Longstanding illness	Yes	24.0	24.3	0.886

The percentages in the table show how much depression-related factors account for the high and low workplace social capital groups

Table 3 shows the association between depression and workplace social capital among men. The OR for depression was higher for low workplace social capital. The adjusted OR for depression with low workplace social capital was 2.93 (95% confidence interval [CI] 2.16–3.98) (model 5). After adjusting for workplace social capital, the strength of the associations between depression and low grade job position (model 1), low job support (model 2), and moderate family–to–work conflict (model3) decreased and also lost significance. The associations between depression and low control at work (model 2), high family–to–work conflict, and moderate and high work–to–family conflict (model 3) were reduced. After adjusting for all variables, significant associations with depression were found for unmarried, low and moderate control at work, high family–to–work conflict, moderate and high work–to–family conflict, sleep of < 6 h, and presence of longstanding illness, while the associations between depression and long work hours and high work demands disappeared (model 5).

**Table 3:** The association between workplace social capital and depression in men

		Age-adjusted ORs	Model 1	Model 2	Model 3	Model 4	Model 5
	Prevalence of depression(%)	(95%CI)	Work life condition (95%CI)	Job stress (95%CI)	Work–life balance (95%CI)	Physical health (95%CI)	Full adjusted (95%CI)
Workplace social capital							
High	17.6	1.00	1.00	1.00	1.00	1.00	1.00
Low	47.3	4.14(3.25–5.38)	4.11(3.18–5.33)	3.39(2.58–4.46)	3.42(2.60–4.49)	4.12(3.18–5.33)	2.93(2.16–3.98)
Age							
20–29	20.9	0.94(0.63–1.42)	0.40(0.24–0.68)	0.99(0.63–1.55)	1.54(0.96–2.45)	1.20(0.77–1.87)	0.85 (0.46–1.57)
30–39	23.8	1.12(0.82–1.53)	0.68(0.45–1.02)	1.04(0.74–1.46)	0.88(0.62–1.24)	1.29(0.91–1.84)	0.83 (0.52–1.32)
40–49	24.2	1.14(0.88–1.46)	0.81(0.59–1.09)	0.96(0.74–1.27)	0.85(0.63–1.13)	1.11(0.84–1.46)	0.78 (0.55–1.11)
50–59	21.9	1.00	1.00	1.00	1.00	1.00	1.00
Marital status							
Married	21.2	1.00	1.00				1.00
Unmarried	30.3	1.92(1.44–2.56)	1.93(1.43–2.62)				2.98 (2.09–4.24)
Job position							
Low grade	25.2	1.73(1.19–2.51)	1.40(0.94–2.09)				1.01 (0.64–1.59)
Middle grade	21.3	1.24(0.86–1.81)	1.14(0.77–1.68)				0.86(0.56–1.32)
High grade	18.3	1.00	1.00				1.00
Work hours							
< 9 h	20.3	1.00	1.00				1.00
9-11 h	25.2	1.33(1.03–1.71)	1.46(1.11–1.91)				0.96(0.70–1.32)
>11 h	36.7	2.33(1.62–3.34)	2.63(1.79–3.87)				1.43(0.89–2.29)
Shift work							
Yes	28.5	1.38(0.95–2.00)	1.47(0.99–2.19)				1.14(0.73–1.77)
No	22.5	1.00	1.00				1.00
Control at work							
Low	35.3	3.17(2.41–4.16)		2.90(2.17–3.88)			2.58(1.87–3.58)
Intermediate	23.5	1.78(1.35–2.35)		1.86(1.40–2.48)			1.60(1.17–2.19)
High	14.7	1.00		1.00			1.00
Demand at work							
Low	15.6	1.00		1.00			1.00
Intermediate	23.4	1.64(1.24–2.18)		1.82(1.35–2.45)			1.35(0.96–1.90)
High	30.3	2.33(1.77–3.08)		2.43(1.81–3.27)			1.15(0.80–1.66)
Support at work							
Low	30.9	2.05(1.55–2.73)		1.19(0.86–1.6)			1.09(0.76–1.54)
Intermediate	21.4	1.23(0.94–1.62)		1.05(0.78–1.40)			1.03(0.75–1.41)
High	18.6	1.00		1.00			1.00
Family–to–work conflict							
Low	11.7	1.00			1.00		1.00
Intermediate	17.9	1.70(1.18–2.46)			1.22(0.83–1.80)		1.34(0.89–2.02)
High	39.4	5.16(3.97–6.71)			2.95(2.21–3.93)		3.38(2.47–4.61)
Work–to–family conflict							
Low	8.6	1.00			1.00		1.00
Intermediate	19.6	2.70(1.91–3.81)			2.08(1.45–3.00)		1.98(1.35–2.92)
High	41.8	8.15(5.81–11.43)			4.81(3.33–6.94)		4.51(2.96–6.86)
Sleep hours							
<6 h	39.6	2.63(2.01–3.45)				2.69(2.02–3.58)	1.76(1.27–2.44)
6-8 h	19.9	1.00				1.00	1.00
≧9 h	35.7	2.24(0.74–6.74)				1.93(0.60–6.21)	1.65(0.44–6.12)
Longstanding illness							
Yes	28.7	1.81(1.43–2.29)				1.79(1.40–2.29)	1.55(1.18–2.05)
No	19.7	1.00				1.00	1.00

Model 1 is adjusted for age, workplace social capital, and work life condition (marital status, job position, work hours, shift work)

Model 2 is adjusted for age, workplace social capital, and job stress (control at work, demand at work, support at work)

Model 3 is adjusted for age, workplace social capital, and work–life balance (family–to–work conflict, work–to–family conflict)

Model 4 is adjusted for age, workplace social capital, and physical health (sleep hours, longstanding illness)

Model 5 is adjusted for age, workplace social capital, work life condition, job stress, work–life balance, and physical health

Table 4 shows the association between workplace social capital and depression among women. The OR for depression was higher for low workplace social capital. The adjusted OR of workplace social capital to depression was 2.46 (95% CI 1.74–3.49) (model 5). After adjusting for workplace social capital, the strength of the associations between depression and unmarried status (model 1) and moderate control at work (model 2) were reduced and lost significance. The associations between depression and long work hours (model 1), low control at work, low and moderate job support (model 2), high family–to–work conflict, moderate and high work–to–family conflict, and sleep of < 6 h were reduced. After adjusting for all variables, significant associations with depression were found for unmarried, low and moderate job support, high family–to–work conflict, moderate and high work–to–family conflict, and sleep periods of < 6 h, while the associations between depression and long work hours, shift work, low control at work, moderate and high work demands, and longstanding illness disappeared (model 5).

**Table 4:** The association between workplace social capital and depression in women

		Age-adjusted ORs	Model 1	Model 2	Model 3	Model 4	Model 5
	Prevalence of depression(%)	(95%CI)	Work life condition (95%CI)	Job stress (95%CI)	Work–life balance (95%CI)	Physical health (95%CI)	Full adjusted (95%CI)
Workplace social capital							
High	24.0	1.00	1.00	1.00	1.00	1.00	1.00
Low	53.3	3.80(2.83–5.11)	3.69(2.73–4.99)	2.85(2.06–3.94)	3.39(2.47–4.65)	3.38(2.73–4.96)	2.46(1.74–3.49)
Age							
20–29	36.4	1.84(1.26–2.70)	1.57(0.93–2.67)	2.64(1.73–4.04)	3.95(2.50–6.22)	2.77(1.80–4.27)	3.33 (1.80–6.15)
30–39	33.2	1.60(1.09–2.35)	1.45(0.90–2.33)	1.80(1.19–2.73)	1.95(1.27–2.98)	2.01(1.32–3.07)	1.99 (1.16–3.41)
40–49	26.5	1.16(0.78–1.74)	1.25(0.80–1.96)	1.36(0.88–2.09)	1.15(0.74–1.79)	1.46(0.95–2.25)	1.32 (0.80–2.18)
50–59	23.7	1.00	1.00	1.00	1.00	1.00	1.00
Marital status							
Married	24.8	1.00	1.00				1.00
Unmarried	37.6	1.67(1.23–2.27)	1.36(0.98–1.89)				2.54 (1.71–3.76)
Job position							
Low grade	31.3	1.41(0.55–3.60)	1.16(0.43–3.11)				1.02 (0.36–2.91)
Middle grade	27.6	1.65(0.62–4.37)	1.33(0.48–3.67)				1.28(0.44–3.74)
High grade	18.2	1.00	1.00				1.00
Work hours							
< 9 h	25.0	1.00	1.00				1.00
9-11 h	35.3	1.62(1.23–2.13)	1.47(1.10–1.98)				1.11(0.79–1.57)
>11 h	46.2	2.41(1.58–3.69)	2.15(1.37–3.37)				1.33(0.80–2.22)
Shift work							
Yes	36.1	1.51(1.17–1.96)	1.34(1.01–1.77)				1.07(0.78–1.47)
No	26.1	1.00	1.00				1.00
Control at work							
Low	37.5	2.00(1.44–2.77)		1.58(1.10–2.26)			1.45(0.98–2.14)
Intermediate	30.6	1.44(1.04–2.00)		1.29(0.91–1.82)			1.03(0.71–1.49)
High	23.0	1.00		1.00			1.00
Demand at work							
Low	19.2	1.00		1.00			1.00
Intermediate	31.0	1.88(1.32–2.70)		2.20(1.50–3.22)			1.37(0.89–2.10)
High	37.9	2.63(1.88–3.69)		2.82(1.97–4.03)			1.49(0.97–2.30)
Support at work							
Low	40.7	3.24(2.27–4.61)		2.05(1.37–3.06)			1.97(1.29–3.02)
Intermediate	35.7	2.29(1.69–3.10)		1.83(1.33–2.51)			1.89(1.34–2.66)
High	21.2	1.00		1.00			1.00
Family–to–work conflict							
Low	23.0	1.00			1.00		1.00
Intermediate	22.1	1.01(0.64–1.60)			0.76(0.46–1.25)		0.81(0.48–1.36)
High	40.2	2.89(2.15–3.89)			2.01(1.44–2.79)		2.92(2.00–4.27)
Work–to–family conflict							
Low	10.9	1.00			1.00		1.00
Intermediate	26.0	3.20(2.05–5.02)			2.58(1.62–4.12)		2.38(1.46–3.906)
High	45.5	8.55(5.52–13.23)			6.03(3.80–9.58)		4.82(2.89–8.04)
Sleep hours							
<6 h	43.5	2.23(1.65–3.02)				2.01(1.47–2.75)	1.60(1.13–2.27)
6-8 h	27.0	1.00				1.00	1.00
≧9 h	40.0	1.60(0.44–5.78)				1.52(0.40–5.75)	1.40(0.33–5.91)
Longstanding illness							
Yes	33.3	1.47(1.08–2.00)				1.50(1.08–2.08)	1.28(0.90–1.83)
No	29.6	1.00				1.00	1.00

Model 1 is adjusted for age, workplace social capital, and work life condition (marital status, job position, work hours, shift work)

Model 2 is adjusted for age, workplace social capital, and job stress (control at work, demand at work, support at work)

Model 3 is adjusted for age, workplace social capital, and work–life balance (family–to–work conflict, work–to–family conflict)

Model 4 is adjusted for age, workplace social capital, and physical health (sleep hours, longstanding illness)

Model 5 is adjusted for age, workplace social capital, work life condition, job stress, work–life balance, and physical health

Table 5 shows the effect modification by workplace social capital in the association between depression and work–life condition, job stress, work–life balance, and physical health. In men, after stratification with workplace social capital, the variables with higher odds ratios for depression in the lower workplace social capital group were unmarried status, 9–11 h of work, low/moderate job control, high work–to–family conflict, < 6 h of sleep, and longstanding illness. In women, after stratification with workplace social capital, the variables with higher odds ratios for depression in the low workplace social capital group were ≥ 9–11 work hours, moderate / high job requirements, and moderate/high work–to–family conflict.

**Table 5:** Effect modification by workplace social capital on the association between depression and workplace and family stress

	Men			Women		
		Stratified by Workplace social capital			Stratified by Workplace social capital	
	All	High	Low	All	High	Low
	OR (95%CI)	OR (95%CI)	OR (95%CI)	OR (95%CI)	OR (95%CI)	OR (95%CI)
Marital status						
Married	1.00	1.00	1.00	1.00	1.00	1.00
Unmarried	1.92(1.44–2.56)	1.79(1.26–2.54)	2.39(1.32–4.33)	1.67(1.23–2.27)	1.55(1.06–2.27)	1.38 (0.77–2.48)
Job position						
Low grade	1.73(1.19–2.51)	1.30(0.83–2.04)	1.56(0.70–3.47)	1.41(0.55–3.60)	1.66(0.47–5.91)	0.29 (0.03–2.95)
Middle grade	1.24(0.86–1.81)	1.12(0.72–1.75)	1.05(0.46–2.36)	1.65(0.62–4.37)	2.24(0.61–8.22)	0.30(0.03–3.31)
High grade	1.00	1.00	1.00	1.00	1.00	1.00
Work hours						
< 9 h	1.00	1.00	1.00	1.00	1.00	1.00
9-11 h	1.33(1.03–1.71)	1.27(0.93–1.73)	1.91(1.12–3.34)	1.62(1.23–2.13)	1.49(1.07–2.08)	2.05(1.19–3.54)
>11 h	2.33(1.62–3.34)	2.41(1.56–3.71)	1.94(0.93–4.04)	2.41(1.58–3.69)	2.19(1.31–3.66)	3.03(1.24–7.42)
Shift work						
Yes	1.38(0.95–2.00)	1.23(0.77–1.96)	1.52(0.72–3.18)	1.51(1.17–1.96)	1.60(1.16–2.20)	1.20(0.73–1.98)
No	1.00	1.00	1.00	1.00	1.00	1.00
Control at work						
Low	3.17(2.41–4.16)	2.53(1.82–3.52)	3.34(1.93–5.79)	2.00(1.44–2.77)	1.48(0.99–2.20)	1.90(0.96–3.77)
Intermediate	1.78(1.35–2.35)	1.60(1.16–2.21)	2.22(1.24–3.97)	1.44(1.04–2.00)	1.28(0.88–1.87)	1.68(0.80–3.51)
High	1.00	1.00	1.00	1.00	1.00	1.00
Demand at work						
Low	1.00	1.00	1.00	1.00	1.00	1.00
Intermediate	1.64(1.24–2.18)	1.61(1.14–2.27)	1.75(0.98–3.11)	1.88(1.32–2.70)	1.72(1.12–2.65)	2.89(1.41–5.92)
High	2.33(1.77–3.08)	2.23(1.60–3.12)	2.01(1.16–3.48)	2.63(1.88–3.69)	2.23(1.48–3.36)	3.63(1.91–6.88)
Support at work						
Low	2.05(1.55–2.73)	1.20(0.83–1.74)	1.55(0.82–2.93)	3.24(2.27–4.61)	2.29(1.42–3.70)	1.48(0.72–3.02)
Intermediate	1.23(0.94–1.62)	1.20(0.88–1.63)	0.77(0.39–1.53)	2.29(1.69–3.10)	2.11(1.49–2.99)	1.43(0.69–2.96)
High	1.00	1.00	1.00	1.00	1.00	1.00
Family–to–work conflict						
Low	1.00	1.00	1.00	1.00	1.00	1.00
Intermediate	1.70(1.18–2.46)	2.10(1.37–3.23)	0.70(0.33–1.48)	1.01(0.64–1.60)	1.19(0.68–2.09)	0.58(0.25–1.35)
High	5.16(3.97–6.71)	4.86(3.54–6.69)	3.92(2.32–6.61)	2.89(2.15–3.89)	3.14(2.17–4.54)	2.50(1.41–4.42)
Work–to–family conflict						
Low	1.00	1.00	1.00	1.00	1.00	1.00
Intermediate	2.70(1.91–3.81)	2.57(1.71–3.86)	2.64(1.30–5.35)	3.20(2.05–5.02)	2.78(1.67–4.60)	3.29(1.11–9.80)
High	8.15(5.81–11.43)	6.58(4.42–9.78)	10.89(5.27–22.48)	8.55(5.52–13.23)	6.56(4.03–10.69)	11.54(3.87–34.40)
Sleep hours						
<6 h	2.63(2.01–3.45)	2.39(1.73–3.32)	3.81(2.06–7.04)	2.23(1.65–3.02)	2.48(1.70–3.60)	1.30(0.74–2.29)
6-8 h	1.00	1.00	1.00	1.00	1.00	1.00
≧9 h	2.24(0.74–6.74)	2.24(0.57–8.78)	1.87(0.25–13.94)	1.60(0.44–5.78)	2.51(0.55–11.58)	0.42(0.04–4.85)
Longstanding illness						
Yes	1.81(1.43–2.29)	1.44(1.07–1.92)	3.11(1.92–5.05)	1.47(1.08–2.00)	1.57(1.08–2.29)	1.43(0.77–2.68)
No	1.00	1.00	1.00	1.00	1.00	1.00

*OR* odds ratio for depression adjusting for age, *CI* confidence interval

The Hosmer–Lemeshow test [32] validated the final models (Model 5 in Tables 3 and 4). The interaction terms of any 2 variables for work–life condition, job stress, work–life balance, and physical health did not add significantly to the models.

## Discussion

In this study, workplace social capital was independently associated with depression among the adjusted variables (work–life condition, job stress, work–life balance, and physical health). Workplace social capital mitigated the associations between depression and stresses from work, home, or physical health in both men and women.

In terms of the association between workplace social capital and each variable, men and women who had low control at work, high work demands, low work support, and high work–to–family conflict felt significantly less workplace social capital. A high proportion of men who had a low job position or longstanding illness and women who were unmarried or sleeping less felt significantly less workplace social capital. This suggests that job stress and work–life balance are strongly related to workplace social capital in both men and women.

For both men and women, the OR of low workplace social capital for depression was significantly higher; (adjusted OR, 2.93 [95% CI 2.16–3.98]) for men (adjusted OR, 2.46 [95% CI 1.74–3.49]) for women. Previous studies have examined the association with depression in men, adjusting for variables of work–life condition, job stress, work–life balance, and physical health, and the OR between depression and low job status was still significant [34, 35]. However, when workplace social capital was added to those variables, as in the present study, the OR between depression and low job status was no longer significantly different. One of the factors that may have contributed to this finding was the fact that men held higher job statuses than women in this study. When one’s position, or job status is higher, one has to organize the workplace and produce good work; however, with a lack of workplace social capital, it would be harder to improve work performance, which could increase depression.

Although workplace social capital and depression showed a strong association with workplace social capital, after adjusting for all variables, long hours and high job demands were no longer associated with depression for both men and women. This confirms the results of previous studies [6–8], which suggested that the cause of workplace depression is not overworked but rather the work environment. The associations with depression were reduced for both men and women, but significant associations remained including high family–to–work conflict, moderate and high work–to–family conflict, and sleep of < 6 h.

Regardless of high or low workplace social capital, home–work/work–family conflict and short sleep duration were strongly associated with depression. The OR of depression to family–to–work conflict was not reduced, especially among women. A previous study reported poor work–life balance, especially among Japanese women [36]. This may be an indication that women play many central roles in the family, including child-rearing and household chores. The association between depression and longstanding illness remained significantly different for men, whereas the significance of the association with longstanding illness disappeared for women. We hypothesized that because men were older than women in the target population, physical health was more likely to affect mental health in men than in women in this study; thus expecting the percentage of people with longstanding illness to increase with age. In contrast, the association between depression and being unmarried was enhanced for both men and women after adjusting for all variables. Being married has been reported to contribute to greater mental stability [29]. It was considered that being married buffers the negative effect of lack of social capital at work and thereby reduces the risk of depression.

A difference was found between the genders for the variable of job stress. For men, control at work was an important factor in managing stress, whereas for women, job support was more significant. It was inferred that for men, an environment in which they could work under their own control was desirable for their mental health, while for women, an environment in which they could receive substantial work support from their colleagues at work was desirable.

When stratified by high and low workplace social capital, the ORs of long working hours and work–to–family conflict for depression were higher in the low workplace social capital group for both men and women. This suggests that workplace social capital may mitigate work-related stress and reduce the risk of depression.

The association between depression and job status, job stress, and poor work–life balance was mitigated by workplace social capital, indicating that fostering workplace social capital may reduce or prevent the risk of depression. By gender, workplace social capital reduced the association of depression with low job position and low job support, particularly for men. For women, workplace social capital reduced the association between depression and being unmarried and working shifts. Because men and women may differ in their positions at work and at home, the roles they play, and their desired work environment, it is possible that a work environment that includes workplace social capital according to gender characteristics, i.e., a workplace where people understand and acknowledge each other, where men can get a high control at work, and where women can get actual job support, may be effective in maintaining and improving the mental health of workers.

There are several limitations to this study. First, this is a cross-sectional study and it is not possible to rule out the possibility of reverse causation, i.e., people with depression tend to feel that they have low workplace social capital. However, a prospective cohort study of Finnish public sector workers reported that there was a significant association between low workplace social capital and depression, even after adjusting for psychological distress at baseline [18]. Because of the complex relationship between depression and workplace social capital, work–life condition, job stress, work–life balance, and physical health, future longitudinal studies should be conducted to confirm these relationships. Second, there is a risk of common method bias in the data for this study [37]. Since multiple variables were taken from the same respondent in this study, it is possible that the subject was trying to maintain consistency and showed a higher correlation than the true correlation. Furthermore, subjects may have responded in a socially desirable manner due to their position as civil servants. Third, the subjects were civil servants, which may not be representative of the mental health and workplace social capital of the general workforce. The mean workplace social capital of the study’s subjects (2.98 SD 0.68 points for men and 2.91 SD 0.68 points for women) was better than the national mean for both men and women (2.58 SD 0.76 points for men and 2.75 SD 0.76 points for women) [38], suggesting that they are a group with high workplace social capital. Even with these limitations, we believe the results of this large survey show that workplace social capital can independently reduce depression, even when adjusted for work and family stress.

## Conclusions

Workplace social capital for both men and women showed independent associations between depression and workplace variables; however, workplace social capital reduces the association between depression and work–life condition, job stress, work–life balance, and physical health. Although future longitudinal studies are needed to ascertain the relationship between depression and workplace social capital, work situation, work and family stress, and physical health, it may be possible to reduce or prevent the risk of depression in workers by creating a working environment that fosters workplace social capital.

## Data Availability

The datasets analyzed during the current study are available from the corresponding author on reasonable request.

## Abbreviations

SES: Socioeconomic status
CI: Confidence interval
OR: Odds ratio
